# What does quality of care mean for maternal health providers from two vulnerable states of India? Case study of Bihar and Jharkhand

**DOI:** 10.1186/s41043-016-0043-3

**Published:** 2016-02-20

**Authors:** Shilpa Karvande, Devendra Sonawane, Sandeep Chavan, Nerges Mistry

**Affiliations:** Foundation for Research in Community Health, Pune, India

**Keywords:** Healthcare quality, Maternal-child health services, Quality perspectives

## Abstract

**Background:**

Quality instillation has its own challenges, facilitators and barriers in various settings. This paper focuses on exploration of quality components related to practices, health system challenges and quality enablers from providers’ perspectives with a focus on maternal health studied through a pilot research conducted in 2012–2013 in two states of India—Bihar and Jharkhand—with relatively poor indicators for maternal health.

**Methods:**

Qualitative data through in-depth interviews of 49 health providers purposively selected from various cadres of public health system in two districts each from Bihar and Jharkhand states was thematically analysed using MAXQDA Version 10.

**Results:**

Maternity management guidelines developed by the National Health Mission, India, were considered as a tool to learn instillation of quality in provision of health services in various selected health facilities. Infrastructure, human resources, equipments and materials, drugs, training capacity and health information systems were described as health system challenges by medical and paramedical health providers. On a positive note, the study findings simultaneously identified quality enablers such as appreciation of public-private partnerships, availability of clinical guidelines in the form of wall posters in health facilities, efforts to translate knowledge and evidence through practice and enthusiasm towards value of guidelines.

**Conclusions:**

Against the backdrop of quality initiatives in the country to foster United Health Care (UHC), frontline health providers’ perspectives about quality and safety need to be considered and utilized. The provision of adequate health infrastructure, strong health management information system, introduction of evidence-based education and training with supportive supervision must constitute parallel efforts.

## Background

Globally, the maternal mortality ratio (MMR) has declined by 47 % over the past two decades with the highest reduction in Eastern Asia (69 %) and Southern Asia (64 %). In India, the National Rural Health Mission (NHM) launched by the Government of India is a leap forward in establishing effective integration and convergence of health services and affecting architectural correction in the healthcare delivery system in India [1]. It has developed a series of guidelines and has launched various programmes and initiatives such as the Janani Suraksha Yojana, the Janani Shishu Suraksha Karyakram and the Reproductive Maternal Neonatal Child and Adolescent Health (RMNCH+A) [2] for improving maternal health and quality of care. Additionally, it has set the Indian Public Health Standards (IPHS) for standardized service provision. However, the unfinished agenda of maternal and child mortality still exerts immense strain on the overstretched health systems in discrete locations.

Furthermore, the dichotomy in the health performance of various states of India draws attention towards state-specific needs and priorities. The state of Kerala with an MMR as low as 61 [3] is striving to bring it further down, whereas vulnerable states such as Bihar [2] and Jharkhand [3] with MMR of 274 and 245, respectively, are struggling to reach the national MMR figure of 167 [3]. Besides, MMR percentage of home deliveries (Kerala—0.0, Bihar—42.1, Jharkhand—53.4) and unmet need of family planning (Kerala—19 [4], Bihar—31.5 and Jharkhand—22.3 [5]) highlight state disparity.

A systematic literature review showed that there is no universally accepted definition of quality of care which is widely accepted as multifaceted [6]. Outcomes of pregnancy and utilization of maternal healthcare services are studied to reflect lack of quality. Various studies [7–11] have highlighted health system challenges in terms of delay in obtaining obstetric care, service provisioning, access and cultural issues. Herein, fragmented and unregulated health-care delivery systems, poor availability of trained human resources for health and social determinants of health have been identified as some of the issues to be addressed for achieving universal health coverage by 2022 [12]. Most of the studies have addressed users’ perspectives regarding health system challenges or are based on empirical evidence collected by researchers. Relatively less is known about the perspectives and practices of health providers at multiple levels even in international literature. Two studies present provider perspectives to understand health system challenges [13, 14]. Provider perspectives are important since they impinge on adherence to quality standards, professional team work, quality and training of medical education and interaction with the communities that are served by them.

Achieving quality maternal health care is the ultimate desired outcome at the national level; however, the strategies to achieve the outcome need to have state-specific contexts. This paper focuses on case studies of two vulnerable states—Bihar and Jharkhand—conducted in 2012–2013 based on exploration of quality components related to practices, services, systems and human resources from providers’ perspectives with a focus on maternal health.

## Methods

The pilot research was conducted in two selected states—Bihar and Jharkhand (two districts each)—during October 2012–May 2013. During the initial visits, a study team of two public health researchers interacted with local key organizations to understand maternal health situation in the state and to learn specific characters of districts. The state governments were approached for seeking formal approval to conduct the research. The district selection was governed by district statistics for maternal health, logistic feasibility for data collection and suggestion from the state government. Attempts were made to include two contrasting districts from each district. Healthcare providers primarily from various health cadres in the public health sector formed the sample for this study.

### Selected states and districts

Bihar has 38 districts with a total population of 103 million. The state has launched several initiatives, mostly in collaboration, such as the Family Friendly Hospital Initiative under the SWASTH programme [15] focusing on patient care, patient safety, patient stay and patient feedback and the Ananya programme with the Bill and Melinda Gates Foundation with an objective to ensure that mothers and babies survive and remain healthy during pregnancy, childbirth, and early childhood. Additionally, there are public-private partnership (PPP) schemes for service provisions. Bihar has implemented HIS strengthening project in collaboration with UNFPA since October 2009 [16].

Jharkhand state from eastern India was carved out of the southern part of Bihar in November 2000. The state has a population of 32 million residing in 24 districts. The state implements several collaborative initiatives such as the MCHIP programme of USAID for implementation of the RMNCH+A approach for reduction in mortality through focused maternal and child health interventions or UNICEF initiative focusing on child health and immunization, skilled birth attendance and basic emergency obstetric care (BEmOC). The state has initiated implementation of Electronic Health Management Information System (e-HMIS) in 2012 (Table 1).

**Table 1:** Key maternal health indicators for selected states of Bihar and Jharkhand

Health indicator	Bihar	Jharkhand
Total population (million) [3]	104	32.9
Decadal growth (%) [3]	25	22.34
Crude birth rate [5]	26.1	23
Natural growth rate [5]	19.3	18.1
Sex ratio [3]	918	947
Child sex ratio [3]	935	943
Infant mortality rate [5]	48	36
Maternal mortality ratio [5]	274	245
Total fertility rate [5]	3.5	2.7
Percentage of women with institutional delivery [5]	55.4	46.2
Percentage of women who had delivery at home [5]	42.1	53.4
Home delivery assisted by skilled persons [5]	30	27.4
Percentage of safe delivery [5]	64.5	56.2
Mothers who had three or more ANC [5]	36.7	60.2

### Health facilities

All Indian states have a three-tiered rural healthcare system with the health sub-centre (HSC) as the most peripheral contact point generally serving a population of 5000. The primary health centre (PHC) is a referral unit for about six sub-centres with four to six beds generally serving a population of 30,000. The third tier—Community Health Centre (CHC)—is a 30-bedded referral hospital with four PHCs and having specialized services, generally serving a population of 120,000 [17]. In Bihar and Jharkhand, few health sub-centres were upgraded to serve as delivery points aiming to cater for maternity services. Personnel from health facilities at each of the three levels from the selected districts were included in the pilot research for understanding quality perspectives and health system challenges (Table 2).

**Table 2:** Profile of health facilities (*n* = 32) visited during the study

Type of health facilities visited during the study	Bihar (*n* = 17)			Jharkhand (*n* = 15)		
	Public	Private	Informal	Public	Private	Informal
District hospitals	2	2	1	2	3	1
Sub-district/referral hospitals	2			0		
Block level hospitals	3			3		
Peripheral hospital-primary health centres/health sub-centres	6			6		
ANM training school	1					

The district health teams as well as local NGOs helped the research team for planning the field work.

### Profile of the respondents

**Fig. 1:**
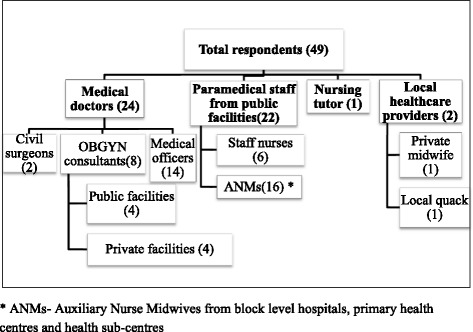
Profile of respondents based on their designation

In all 49 health providers based at 32 selected health facilities from the two states were interviewed. They included doctors, nurses (staff nurses and auxiliary nurse midwives), private practitioners and academicians from formal public health sector, and informal health providers (quacks) involved in provision of maternal health services were also interviewed. The selection of health providers was purposive depending upon their involvement in provision of maternal health care in the selected district and availability at the time of interview (Fig. 1).

### Study tool

The project focused on the following domains of maternal healthcare, viz. antenatal care, delivery practices, intra and post-natal care and family planning. Data was primarily derived from in-depth interviews of health providers in English and Hindi (for nurses). An interview guide was designed with the following themes: (a) clinical practices related to maternity management, (b) experience of provision of maternal health care including challenges, and (c) perceptions regarding guideline-based management and quality enablers.

Additionally, field observations made by the study team supported the primary data collected from the interview, for instance, number of beds in the health facility or the functionality of an intensive care unit.

### Ethics considerations

The research proposal received ethics clearance from the Institutional Research and Ethics Committee of Foundation for Research in Community Health (IREC/2012/22/9). Written permission to conduct this research was obtained from the state health officials of the respective state. Each respondent was interviewed one-on-one at the health facility, subsequent to seeking written informed consent for conducting and audio-recording of the interview. Privacy during interview, anonymity and confidentiality of information shared were strictly maintained. At times, additional precaution had to be taken during the interviews of paramedical staff to avoid interference from their senior staff to influence their responses.

### Data management and analysis

Qualitative data were transcribed and translated in English, wherever necessary. Thematic codes—inductive and deductive—were generated, viz. key clinical practices, perceptions of quality, health infrastructure challenges, community dynamics and attitude towards guidelines. The data were processed with the software MAXQDA Version 10.

## Results

The results are presented under three categories—practices in maternity management, health system challenges and quality enablers.

### Practices in maternity management

Maternity management guidelines developed by NHM were considered as a standard of quality for provision of health services in various selected health facilities. Respondents were asked about practices for provision of key components of maternity management including routine antenatal care investigations, the use of partograph for monitoring progress of labour and management of obstetric complications. The medical doctors and nurses reported several deviations from NHM guidelines in terms of non-compliance with the recommended investigations and examinations. Three key variations which emerged were as follows:

A) “Non”-use of partograph—partograph is a composite graphical record of key data (maternal and foetal) during labour entered against time on a single sheet of paper and provides progress of labour. One of the key messages regarding “Care during labour and delivery” in the “SBA Guidelines for Skilled Attendance at Birth” [17] states that “partograph will help the nurse recognize the need for action at the appropriate time and thus ensure timely referral”.. Relevant measurements include cervical dilation, foetal heart rate, duration of labour and vital signs. The nurses mentioned about not using the partograph because of various reasons such as overload of women in labour or nurses perceived insignificance of partograph.
  Partograph is not so useful because in most of the primi cases it suggests referral, but actually it is not so. We cannot refer them in real life situation due to delay and poor access and the woman delivers here. ANM, CHC, Jharkhand

B) Early augmentation of labour at the time of delivery—the training curriculum for skilled birth attendants does not recommend the use of drugs for augmentation of labour in case of normal labour [17]. However, the health providers frequently mentioned about community dynamics as one of the major reasons for early augmentation of labour.
  When the woman comes with labour pains, she is in a hurry. The attendant also feels like getting it over fast. Oxytocin dilates the cervix and is used for augmentation. Medical doctor, block PHC, Bihar
  Subsequently, the NHM guideline about post-natal stay at health facility to be at least of 48 h was also compromised due to reasons such as insufficient beds or lack of warmers during the winter season.

C) No screening for HIV during pregnancy—facility for HIV testing as a desirable component of ANC investigation needs to be available at the CHC [17]. However, HIV testing was not routinely practiced either due to unavailability of laboratory equipments and/or trained human resources.
  We have the necessary testing kits. Our lab technician is trained for HIV testing, but he has forgotten everything. We do not conduct any HIV testing here, but only prescribe the same. Medical officer, CHC, Jharkhand

Deviations in practices related to maternity management were the overall result of the lack of knowledge about the guideline recommendations, skill or attitude of a health provider, perceived community pressure or infrastructural challenges in implementation of the recommendations.

### Health system challenges

Technical and non-technical components viewed as health system challenges by medical and paramedical health providers from the visited public health facilities are as follows:

#### Inadequacy of quality infrastructure

The reference point for gauging infrastructure was the IPHS promoted by the NHM [18]. Bihar and Jharkhand have poor distribution of health facilities, including First Referral Units (FRU). Some of the block PHCs have excessive population coverage and cater to a population of more than 0.2 millions. As per the policy, one block PHC caters to a population of 80,000 to 120,000 [19]. Health providers highlighted challenges in terms of inadequacy of health infrastructure including physical infrastructure, equipments, materials and poorly functioning transport. About 75 % of the health facilities, including the district hospitals, had shortage of beds, at times resulting in patients being required to lie on the floor. The supply of adequate electricity and running water was observed only in 50 % of the visited health facilities. Non-availability of ultrasound machines at the sub-district, referral and even district hospitals compromised early identification of high-risk pregnancies. This was in contravention to the *Operational guidelines for quality assurance in public health facilities* [20]

If we cannot manage the eclampsia patients here, we refer them to Patna [neighbouring district]. If on assessment we know the woman has PROM [Premature Rupture of Membrane] or foetal distress then we will not open her here. We do not have the facilities here. If she developed some respiratory problem, there is no ventilator support here. Obstetrician, sub-district hospital, Bihar

Poor availability of treatment for eclampsia in public facilities forces women to seek eclampsia care mostly from the private sector and incurs heavy out of expenditure [21].

Forty percent of the visited health facilities did not have a regular supply of essential drugs such as folic acid tablets as was mentioned by the health providers and/or observed by the researchers in the study team. Shortage of contraceptives, IFA tablets and pregnancy testing kits [22] and drugs for treatment of eclampsia certainly affects preparedness of health providers to manage pregnancies or emergency obstetric care.

The district societies are supported with funds for procurement of drugs; however it was noticed that the funds are underutilized. State level health official, Bihar

Medicines are not available so we cannot provide the services. What is point in talking about protocols or guidelines? Medical officer, PHC, Bihar

Non-availability of drugs either deprived the women from availing the necessary drugs or increased their out-of-pocket expenditure since they were asked to procure the drugs from private drug stores.

Guideline implementation for referral of obstetric cases with high risks or complications can only be effected with functional transport arrangements. Both states have a toll-free facility of ambulance referred as “108” which works exclusively for transport of women in labour to hospital and to their homes, 48 h after delivery. However, an occasional breakdown of these vehicles and unduly extended repair was a problem in the peripheral health facilities.

The ambulance had broken down for one month. Local leaders even got up to beating us. If we do something today and cannot offer it tomorrow, my complaint will go to the higher authority. Because of this fear, we cannot start anything. Medical officer, PHC, Bihar

Inadequate EmOC services in both the states especially Jharkhand further compromised quality of maternal health care. This was reflected both in shortage of trained staff at even as high as the district level hospital and lack of ultra sonography facilities as cited by the health providers and observed during the study visits. Subsequently, referral cases either had to visit private facilities or were managed by other informal health providers including local quacks. There were also incidents of involvement of local quacks for unnecessary augmentation of labour. The study team approached one such quack in Jharkhand. He was an agriculturist settled down in one of the study districts and provided medical aid upon demand from a community starved of formally trained providers.

I inject drug Syntecinone to augment labour, on demand from the community. Local quack, Jharkhand

#### Shortage of trained workforce

About 90 % of the visited health facilities reported a problem of managing workload against the available trained manpower due to vacancies in the posts, e.g. one of the districts of Bihar had 27 medical doctors posted against the sanctioned number of 106 [23]. In general, there are significant vacancies for doctors, nurses and paramedical staff in Bihar [22].

A shortage of specialists was a problem even at the district hospital level.

When a baby is premature and critical, we refer. We do have a pediatric ICU here, but there is no pediatrician which makes it difficult to monitor the baby 24 hrs. Obstetrician, district hospital, Jharkhand

In Jharkhand, only one out of ten auxiliary nurse midwife (ANM) training centres were functional. Hence, quality as well as quantity of trained human resource was a challenge for the health system. In recognition of the gaps in pre-service training, the government conducted in-service training for all their staff towards implementation of guidelines. Nevertheless, technical and non-technical (soft) skills were emphasized as gaps in such trainings in these two states.

Necessary things [infrastructure and equipment] are here but we are not trained, for e.g. for laparoscopy, and hence we cannot do it. Also we need to train paramedical staff in order to have significant contribution from them as a team member. Obstetrician, district hospital, Bihar

Nursing staff are trained for various issues such as breastfeeding. However training them for counselling is needed especially to understand community behaviour and to counsel relatives about women’s stay the health facility for two days post-labour. Civil Surgeon, Bihar

Traditional birth attendants (TBA) locally referred as “Dais” and other informal healthcare providers played a crucial role because they were available in provision of accessible neighbourhood maternity management services. Adding value to their role by orientation and training towards safe delivery was considered as a needed approach for ensuring safe maternity management practices by both local NGOs and private practitioners.

We can train Dais (TBAs) and community health workers in basic and routine health checkups. They can be taught and trained regards to risk factors, warning sign, etc and design tools around that. Private obstetrician, Jharkhand

#### Challenges in translating knowledge into practice

Translating knowledge or recent evidence into practice has multiple dimensions such as knowledge based on needs, field level challenges, perceived importance of experience-based learning over knowledge from books and value of guideline-based management. Ninety percent of the medical doctors and obstetricians interviewed in this study mentioned about following text books in their medical education. The obstetricians were aware about the availability of guidelines for management of obstetric complications, the need for evidence and their updating through attending conferences and personal readings.

Guidelines are changed. RCOG guideline, for example, changes [gets updated regularly]. We follow the same here. Obstetrician, Bihar

The experience of providers was simultaneously mentioned as a crucial guiding factor.

Earlier there used to be trained traditional birth attendants [dai]. These Dais would know about conducting deliveries. They used to tell us, teach us. Experience is the big thing, not only the knowledge from the book. We need to have experience. Medical officer, Bihar

There is no fixed protocol in medical science. Patient is there with you and with your experience and knowledge, you have certain judgment. Patient should be safe as soon and as much as possible. Obstetrician, Bihar

Though there was awareness about recent evidence and guidelines among the obstetricians from both states, a clear preference for experience over evidence-based clinical management was perceived.

#### Perceived community dynamics

Managing community dynamics was equally challenging for healthcare providers while attempting to deliver quality services. The study highlighted community dynamics affecting delivery of quality services, as perceived by the health providers. The health providers mentioned poor awareness among community about the importance of post-natal care. As per the Indian guidelines [17], the duration of post-natal stay should be of 48 h. The doctors and nurses frequently mentioned about non-compliance with this recommended duration of post-natal stay at health facility because of the pressure from the family for early discharge of the woman from the health facility. The providers felt that the early exit of the new mother from the health facility was due to the prevalent unfriendly atmosphere and poor infrastructure. This was supported also by observations by the study researchers.

The language divide between the providers and users were barriers in the maintenance of a friendly atmosphere. It further resulted in gaps in health education and counselling particularly towards preventive aspects of maternity management.

We face some language problems so we are not able to have much communication with the women on issues such as their diet during pregnancy. Medical doctor, CHC, Jharkhand

At times, the reluctance of these communities towards the use of formal public health services proved to be a great challenge in provision of maternal health services. “The importance of efforts to take the services to their doorstep” was mentioned to be the key strategy to improve uptake of services by one of the district health officials from Jharkhand.

#### Lack of a robust HMIS

Both states claimed to have developed a robust HMIS; however, its implementation was observed to be rudimentary. The electronic HMIS was still in its infancy stage in both states.

Data entry by ANMs is a challenge. There is only one data entry operator at a block level generally catering a population of about 200,000. Internet connectivity is poor. There is reluctance from medical doctors for any type of computerized monitoring. HMIS expert at the State level, Bihar

Additionally, there was a lack of analytical capacity for translation of uploaded information into action plans at the district as well as the state level. During the visit, the study team could not observe a functional electronic data upload in any of the peripheral health facilities including primary health centres of the two states. The information regarding referrals between health facilities could not be retrieved by the health officials at the district as well as sub-district level. There were instances of incomplete primary data at sub-centre level, e.g. a blank column of information regarding PROM for the past 1 year.

### Quality enablers

The study findings simultaneously highlighted quality enabling features in the states.

Both states appreciated the need to partner with the private sector and generated several PPP initiatives. Most of the PPPs focused on provision of services. To illustrate, Bihar initiated formal partnerships with the private sector in the form of outsourcing of services such as pathology centres and hospital maintenance services. These services were provided free of cost to the patients in the public health system; however, there was a need to ensure the availability and accessibility of these services at all the stipulated health facilities. There was state level monitoring of the PPPs through state data centres [24] but its benchmarks were unclear.

Both states initiated programmes and schemes for quality improvement in maternal health such as the creation of delivery points at health sub-centre level for the increase of outreach of obstetric services and focused efforts such as provision of comprehensive care in the areas of RMNCH+A in Jharkhand. As per the central mandate regarding implementation of adolescent reproductive and sexual health strategy, Adolescent Reproductive Sexual Health (ARSH) clinics have been established at the CHCs. Though such a centre was present in one of the CHCs visited during the study, implementation of key features to ensure quality of ARSH services, viz. creating conducive environment in the community, could not be confirmed.

Health facilities from both states displayed wall posters depicting certain treatment algorithms and were considered as an important check-list for the paramedical staff.

Such posters [pointing at the poster on importance of breast feeding within ½ an hour after delivery] are helpful especially for the paramedicals. Medical officer, Block PHC, Bihar

However, it was mentioned that other than the protocols displayed in the form of wall posters, the health providers did not receive any quick reference document as “guidelines” from the public sector.

A section of the medical doctors from Bihar expressed the need for better dissemination of guidelines as even the doctors over and above the nurses were largely unaware about NHM guidelines. Obstetricians from the private sector in Jharkhand highlighted the need to have an apex body for generating guidelines and their local adaptations.

There is no national body or uniform procedure for developing guidelines. Government guidelines are there, but not well circulated especially in the private sector. Further, there is no instruction for how to follow these guidelines, whether it is compulsory or not, what would be the legal aspects of (not) following various guidelines is not clear. Private obstetrician, Jharkhand

The emphasis on the need for well-developed guidelines and their accessibility is a highly positive indication for their acceptance and implementation.

The enthusiasm of health providers especially the young nurses drew special attention during the study. Enthusiasm to acquire knowledge regarding clinical practices and to seek inputs for improvisation in routine clinical skills was appreciable. The aspiration of a young ANM from one of the remote CHCs of Jharkhand about being “perfect in giving the episiotomy cut” was one striking example of expression of yearning for quality by primary healthcare providers.

## Discussion

The utility value of the present study lies in the identification of the perspectives that challenge or facilitate the nesting of quality initiatives in India. These insights gathered from largely challenged settings are valuable since they are likely to be representative of several similar areas within India.

The development of guidelines in India since 2005 has itself been an imperfect exercise [25]. Indian guidelines in the field of maternity management are weak on documentation about the guideline development process, incorporation of patient views, weak emphasis on collection, collation and updating of evidence and formulating recommendations [26]. Additionally, the current study highlights the poor availability of guideline recommendations in the appropriate quick reference formats to the peripheral levels of health providers. No quick response guides are available, and the guidelines in poster formats which paramedical providers find useful may not have been strictly vetted for their quality-based content. Adherence to standard treatment protocols needs improvement across states because they are either not available or poorly followed with no mechanism of quality assurance [27] or quality benchmarks [28].

Good health outcomes require a downstream chain of events beginning with well-developed guidelines. The subsequent vital steps comprise of definition of quality benchmarks or standards that should percolate to providers at all levels and information systems and audits that measure and question health outcomes. In India, HMIS is used more for monitoring of tasks completed by peripheral workers rather than for programme management and designing [29]. Though large volumes of data are recorded, there is poor evidence about the use of this information in decision-making [22] and improvement of service. Facility level audits to analyse elements of causality similar to the set of clinical audits developed by NICE International [30] can also augment HMIS for local and regional improvements and should be adopted as a part of the quality fabric at all facilities. Investment in training for this at both pre and in-service stage is of paramount necessity.

Even if such evidence-based guidelines were made available and the standards defined, well-trained human resource and the lack of adequate infrastructure constitute an obvious potent block in the delivery of quality healthcare [31]. The percentage of shortfall of health facilities in Jharkhand is 35, 66 and 22 at sub-centre, primary health centre and community health centre levels, respectively [27].

Bihar currently has 12 government medical colleges [32] and 18 government ANM training schools [33], whereas Jharkhand has three government medical colleges and ten government ANM training schools [27]. Despite the efforts to increase the number of seats for medical and paramedical courses, the availability of quality trained human resource in the public health sector especially the specialists, MBBS doctors, staff nurses and ANMs is a problem in the state [22]. While in-service training are provided by the states, e.g. Intra Uterine Contraceptive Device (IUCD) training for nurses and Integrated Management of Neonatal and Childhood Illnesses (IMNCI) training for doctors, gaps in clinical as well as communication and counselling skills are recorded particularly among primary care providers. These are crucial especially when providers are expected to deal with tribal and vulnerable population who may be overtly resistant to absorbing health messages and practices. Inbuilt mechanism for training and re-training, supportive supervision and, very importantly, opportunities for knowledge exchange and update are also desirable approaches for inclusion.

In a wider context, the situation on the ground advocates for a timely reform of medical education in India focusing on quality and evidence-based learning. Hopefully, the recent initiative of the Medical Council of India in introducing a competency-based education with learner-centric approaches, integration of ethics, attitudes and professionalism and skill development would address the need [34]. Imparting of communication and behavioural change skills to health providers may be an important incorporation during reforms in medical and paramedical education in both pre and in-service training.

A number of studies in infectious diseases highlight the gap between “know” and “do” with respect to the provider [35]. The inability of education to change provider behaviour may need to be supplemented through more efficient regulation. Clinical governance is the main vehicle for continuously improving quality of care [36] as was acknowledged in the late nineteenth century by the NHS in England. This will be important for adopting universal health coverage as a developmental imperative in India [37].

The current study highlights PPP as one of the potential quality enablers. However, the history of PPP in India with unclear definitions of roles, expectations and poor commitments [38, 39] does not give rise to optimism. It can be used only as a stop gap in a narrow window of time because private participation in public health depends heavily on external funding which drives incentivization inherent in PPP models [39]. The Kerala Government’s partnership with the Kerala Federation of Obstetricians and Gynaecologists for development and utilization of quality benchmarks with inputs from NICE International [28] is an example of well-planned PPP model. Since there has been a systematic commitment from the state for implementing quality, this PPP positively influences the health infrastructure, education and community mobilization for uptake of health services. The accountability for ensuring safe quality care should however ultimately rest with the public health sector.

Community dynamics was mentioned as negatively influencing guideline implementation or quality compliance. How does the doctor continue to exert good practices and good judgement against community demand which may be at variance with good practice? Despite the fact that Kerala has made a firm commitment for quality in healthcare, provider perspectives and community dynamics continue to affect guideline-based clinical practice, e.g. high rate of caesarean section [22]. Community education, provider perspective and quality of provider-patient interaction therefore assume vital importance.

With gaps at multiple levels, the need for strengthening and skill building of human resources for quality healthcare should be afforded the highest priority. Besides the advantages in providing better quality of care, this will also enable the optimal utilization of contemporary point of care technologies poised for health and disease control [40].

## Conclusions

Against the backdrop of quality initiatives in the country to foster United Health Care (UHC), addressing frontline health providers’ perspectives about quality and safety need to be acknowledged, addressed and valued. Provision of requisite services including health infrastructure, strong health management information system, introduction of evidence-based education and training with supportive supervision must be a parallel exercise. Furthermore, the health providers must respect health as a fundamental right and medical errors as immoral, illicit and illegal. If not, quality health care will continue to be a mirage in the absence of motivated human resources.
